# The Current Landscape of Clinical Trials for Systemic Treatment of HCC

**DOI:** 10.3390/cancers13081962

**Published:** 2021-04-19

**Authors:** Friedrich Foerster, Peter Robert Galle

**Affiliations:** First Department of Medicine, University Medical Center of the Johannes Gutenberg University Mainz, D-55131 Mainz, Germany; foerstfr@uni-mainz.de

**Keywords:** liver cancer, drugs, therapy, immunotherapy, combination therapy

## Abstract

**Simple Summary:**

Liver cancer is a life-threatening disease. Apart from surgery and catheter-guided therapies, drugs are a central pillar for its treatment. Clinical trials are research studies that are designed to evaluate the treatment effect of a given drug. Therefore, they are the driving force behind innovation and medical progress. One such innovation in the past years has been immunotherapy, which has become increasingly important for treating cancer. Recently, the first such therapy has been approved for the treatment of liver cancer. Current clinical trials are exploring the benefit of immunotherapy and other therapies for this disease. This article gives an overview of such trials paying attention to the different underlying treatment strategies and the varying clinical settings, depending on the stage of the disease.

**Abstract:**

The clinical development of systemic treatments for hepatocellular carcinoma (HCC) has gained significant momentum in recent years. After the unexpected failure of the phase 3 trials testing the PD1-inhibitors nivolumab and pembrolizumab as monotherapy in advanced HCC, a multitude of trials employing different agents in various combinations and at different disease stages have been initiated. The first positive results reported for the combination of atezolizumab and bevacizumab, as the first line treatment of advanced HCC, will bring lasting change to the management of HCC and has increased the odds of success for alternative combination therapies. This review article seeks to provide clarity on the complex and evolving landscape of clinical trials on systemic treatments of HCC. It covers current trials which test various systemic treatments (i) in the first and second line in advanced HCC, (ii) in intermediate HCC, (iii) as adjuvant as well as (iv) neoadjuvant strategies, and (v) including immune interventions other than immune checkpoint inhibition.

## 1. Introduction

Clinical trials (CTs) are the most important tools to produce evidence that a given treatment provides benefits of some measure to patients with a certain condition. For patients with hepatocellular carcinoma (HCC), past CTs have laid the foundations of today’s treatment algorithm [1,2]. CTs have been central to establishing the use of systemic agents for the treatment of advanced HCC, defining how today’s clinicians approach patients at this stage [3]. In this setting, treatment has a palliative intention. Based on the results of past CTs, patients are treated in multiple lines, if possible, starting with the first treatment as first-line and moving to the next treatment as the second-line upon progression or intolerance. The design of future CTs for systemic treatment of HCC can benefit from this experience, particularly with regard to endpoint selection, stratification variables, and target populations [4].

The very first CT to lay the groundwork of treating HCC with a multi-target receptor tyrosine kinase inhibitor (RTKI) in HCC was the SHARP trial, which proved that sorafenib, which blocks VEGFR, PDGFRα, and RAF kinases, significantly extends progression-free and overall survival in comparison to the placebo (Table 1) [5]. Several subsequent CTs failed to demonstrate a benefit for the tested agent, either as first-line treatment against sorafenib or as second-line treatment against placebo (brivanib [6,7], sunitinib [8], linifanib [9], erlotinib added to sorafenib [10], everolimus [11], tivantinib [12], ADI-PEG 20 [13], doxorubicin added to sorafenib [14], and doxorubicin-loaded nanoparticles [15]). High hopes had been placed on monotherapy with so called immune checkpoint inhibitors because of their impressive results in entities such as melanoma and non-small cell lung cancer, and indeed initial results from the phase 2 trials with the PD1-inhibitors nivolumab and pembrolizumab had been promising and resulted in accelerated approval by the FDA (Table 1) [16,17]. However, the subsequent phase 3 trials with nivolumab as first-line treatment tested against sorafenib and with pembrolizumab as a second-line treatment, compared to the placebo, failed to meet their primary endpoints [18,19].

Despite these setbacks, there have been five successful phase 3 CTs since the SHARP trial (Table 1): The REFLECT trial demonstrated that lenvatinib, which inhibits VEGFRs 1 to 3, FGFRs 1 to 4, RET, KIT, and PDGFRα, is non-inferior to sorafenib in terms of overall survival (OS) [20]. The RESORCE trial showed that regorafenib, which targets VEGF 1 to 3, PDGFR, FGFR, KIT, RET, RAF-1, and BRAF, improves survival in the second-line setting versus the placebo in patients with advanced HCC who had progressed but tolerated a minimum dose of sorafenib (≥400 mg/day for ≥20 of last 28 days of treatment) [21]. Similarly, the CELESTIAL trial established that cabozantinib, which blocks MET, VEGFR 1 to 3, RET, KIT, AXL, and FLT3, improves survival after progression on sorafenib in comparison to placebo [22]. Furthermore, ramucirumab, a monoclonal antibody against VEGFR2 was investigated in the REACH trial [23], where it failed to demonstrate a benefit, but in a subsequent trial concentrating on patients with baseline AFP concentrations ≥400 ng/dL, the REACH-2 trial, it showed improved OS in comparison to placebo in patients who had progressed on sorafenib [24].

The most recent phase 3 CT with a positive result has tested the combination of atezolizumab, an anti-PD-L1 antibody, and bevacizumab, an anti-VEGF antibody, (atezo/bev) as first-line treatment against sorafenib. It demonstrated superior progression-free survival (PFS) and OS for atezo/bev making it the first CT to achieve this since the SHARP trial (Table 1) [25,26]. Atezo/bev has thus become the new standard-of-care as first-line treatment for advanced HCC, and all coming CTs for this indication will need to be measured against this new benchmark.

**Table 1:** Past clinical trials on systemic treatments in HCC that resulted in regulatory approval.

Trial Name	Treatment Arms	Line of Therapy	Primary Endpoint	ORR	PFS	OS
SHARP [5]	Sorafenib vs. Placebo	First	OS	2 vs. 1%	5.5 vs. 2.8 months	10.7 vs. 7.9 months (HR 0.69)
REFLECT [20]	Lenvatinib vs. Sorafenib	First	OS	24.1 vs. 9.2%	7.4 vs. 3.7 months	13.6 vs. 12.3 months (HR 0.92)
IMbrave150 [25,26]	Atezolizumab+bevacizumab vs. sorafenib	First	OS and PFS	29.8 vs. 11.3%	6.8 vs. 4.3 months	19.2 vs. 13.4 months (HR 0.66)
RESORCE [21]	Regorafenib vs. Placebo	Second	OS	11 vs. 4%	3.1 vs. 1.5 months	10.6 vs. 7.8 months (HR 0.63)
CELESTIAL [22]	Cabozantinib vs. Placebo	Second and third	OS	4 vs. 1%	5.2 vs. 1.9 months	10.2 vs. 8.0 months (HR 0.76)
REACH-2 [24]	Ramucirumab vs. Placebo (in patients with AFP > 400 ng/mL)	Second	OS	5 vs. 1%	2.8 vs. 1.6 months	8.5 vs. 7.3 months (HR 0.71)
CHECKMATE 040 [16]	Nivolumab * single arm	Second	ORR	15%	N/A	N/A
KEYNOTE 224 [17]	Pembrolizumab * single arm	Second	ORR	17%	N/A	N/A
CHECKMATE 040 [27]	Nivolumab + ipilimumab * single arm	Second	ORR	32%	N/A	N/A

* Regulatory approval by the U.S. Food and Drug Administration but not the European Medicines Agency. HR, hazard ratio; N/A, not available; ORR, overall response rate; OS, overall survival; PFS, progression-free survival.

## 2. Systemic Treatment in the First- and Second-Line for Advanced HCC

Several treatments are attempting to follow in atezo/bev’s footsteps (Table 2): The combinations of cabozantinib and atezolizumab (COSMIC-312; NCT03755791) [28], lenvatinib and pembrolizumab, an anti-PD1 antibody (LEAP-002; NCT03713593) [29], durvalumab, an anti-PD-L1 antibody, and tremelimumab, an anti-CTLA-4 antibody (HIMALAYA; NCT03298451) [30], nivolumab and ipilimumab, an anti-CTLA-4 antibody (CheckMate 9DW; NCT04039607), camrelizumab (SHR-1210), an anti-PD-1 antibody, and apatinib (rivoceranib), a RTKI that selectively inhibits VEGFR2 (NCT03764293), as well as monotherapy with tislelizumab, an anti-PD-1 antibody (RATIONALE-301; NCT03412773) [31] are currently being tested in phase 3 trials and are similar in design: All take place in the first-line setting and include patients with unresectable HCCs, i.e., the intermediate stage (Barcelona Clinic Liver Cancer [BCLC] B) not amenable to or progressing after loco-regional therapy and the advanced stage (BCLC C). The only exception here is CheckMate 9DW, which is limited to advanced HCC. As for the primary endpoint, the field is mixed: COSMIC-312, LEAP-002 and the camrelizumab plus apatinib trial use PFS per RECIST 1.1 and OS, while HIMALAYA, CheckMate 9DW and RATIONALE-301 use only OS.

Further combination therapies are in an earlier phase of clinical development (phase 1 or 2). Notably, all include a checkpoint inhibitor: Two trials are evaluating the combination of regorafenib and nivolumab; GOING as second-line treatment after progression on sorafenib with regorafenib as monotherapy during the first eight weeks (NCT04170556), and RENOBATE as first-line treatment (NCT04310709). Regorafenib is also being studied in combination with pembrolizumab (NCT03347292) and tislelizumab (NCT04183088). The latter consists of two parts, the first evaluating the safety of regorafenib and tislelizumab, the second testing the efficacy of the combination against regorafenib monotherapy. Cabozantinib is also being studied in combination with pembrolizumab as first-line treatment (NCT04442581). Last but not least, a phase 1/2 trial is assessing the safety of sitravatinib, a RTKI which inhibits the TAM family (TYRO3, AXL and MER), VEGFR2 and KIT, as monotherapy and in combination with tislelizumab, followed by an efficacy evaluation of both the monotherapy and the combination in anti-PD-1/PD-L1 antibody naïve, as well as refractory/resistant HCC (NCT03941873).

Importantly, none of the current phase 3 trials on palliative systemic therapy uses atezo/bev as a comparator, but either sorafenib or lenvatinib.

## 3. Systemic Treatment for Intermediate HCC

The IMbrave 150 trial enrolled patients with unresectable HCC, i.e., BCLC B and C stage (>80% of patients had BCLC C) [25]. Since the proportion of patients with BCLC B stage was fairly small (~15%), it is currently not possible to make a final assessment of atezo/bev’s efficacy in this patient group, particularly in comparison to treatment with transarterial chemoembolization (TACE), the current standard of care. However, the ABC-HCC trial (NCT04803994), a large investigator initiated phase 3b trial testing atezo/bev against TACE, will precisely address this question (Table 3). Furthermore, the RENOTACE trial (NCT04777851), another large investigator initiated phase 3 trial will test the combination of regorafenib and nivolumab against TACE. Both CTs could pave the way for systemic treatment to the BCLC B stage. However, the challenge of designing such CTs lies in the fact that they compare two different treatment modalities. Therefore, the ABC-HCC trial employs a novel kind of primary endpoint coined time to failure of treatment strategy, which measures the time until either treatment (atezo/bev or TACE) is discontinued by the investigator because it has failed. In contrast, RENOTACE is more conservative in this respect and employs PFS per mRECIST. Other CTs testing different systemic treatments against TACE in intermediate HCC will certainly follow in the coming years.

Another possibility to treat intermediate HCC might be adding systemic therapy to TACE. There are currently three phase 3 trials that explore whether such an approach is beneficial (Table 3). The LEAP-012 trial is testing the addition of lenvatinib and pembrolizumab to TACE (NCT04246177) [32], the EMERALD-1 trial the addition of durvalumab with or without bevacizumab to TACE (NCT03778957), and the CheckMate 74W trial the addition of nivolumab with or without ipilimumab to TACE (NCT04340193)—all in comparison to TACE alone.

## 4. Adjuvant Systemic Treatment

As of now, there is no systemic treatment with a proven benefit in the adjuvant setting after curative hepatic resection or ablation. The STORM trial had failed to demonstrate a benefit of sorafenib in this regard [33]. Four phase 3 trials that are exploring new approaches to improve the outcome after curative surgery or ablation are currently ongoing (Table 4): The CheckMate 9DX trial is testing adjuvant treatment with nivolumab (NCT03383458) [34], the KEYNOTE-937 trial adjuvant treatment with pembrolizumab (NCT03867084) [35], the IMbrave050 trial adjuvant treatment with atezolizumab and bevacizumab (NCT04102098) [36], and the EMERALD-2 trial adjuvant treatment with durvalumab with or without bevacizumab (NCT03847428) [37]. Among this group of CTs, the IMbrave050 stands out, as its control arm does not include placebo, but only active surveillance.

## 5. Neoadjuvant Systemic Treatment

In the past, systemic treatment did not play a relevant role in the neoadjuvant setting. Therefore, there are currently no mature data supporting the use of systemic agents before surgery or locoregional treatment. However, the first early phase CTs assessing neoadjuvant systemic treatment are currently being conducted (Table 5): The NIVOLEP trial is assessing the efficacy of nivolumab treatment before and after electroporation (NCT03630640); the CaboNivo trial is evaluating the safety of the combination of cabozantinib and nivolumab before hepatic resection in locally advanced/borderline resectable HCC (NCT03299946); the efficacy of pembrolizumab before and after curative ablation or resection is about to be explored (NCT03337841); and the PLENTY202001 trial is testing the efficacy of the combination of lenvatinib and pembrolizumab before liver transplantation in HCC exceeding the Milan criteria (NCT04425226). Several aspects about the latter trial are remarkable: Its control arm stipulates no intervention, while being on the waiting list for a liver transplant. Additionally, it allows for enrolling patients with impaired liver function (up to Child Pugh B7), which is uncommon, since most major CTs limit enrolment to patients with normal liver function (Child Pugh A). The use of immune checkpoint inhibitors in the transplant setting is generally controversial, since such treatment may cause allograft rejection with a potentially fatal outcome [38]. In fact, this is the reason why CTs involving checkpoint inhibitors typically exclude patients who have previously received a solid organ transplant. In this respect, the allograft rejection rate in the treatment group will be very informative.

All in all, the neoadjuvant setting is an uncharted territory for systemic treatment and it remains to be seen which of these novel concepts will finally mature into clinical practice.

## 6. Systemic Treatment beyond Immune Checkpoint Inhibition

The inhibition of the so called immune checkpoints—and of PD1/PD-L1 and CTLA-4 in particular—is currently the mainstay of cancer immunotherapy. However, there are many more targets that can potentially be exploited by different immune interventions. Such interventions hold the promise to be effective in patients that are or have become resistant to classical immune checkpoint inhibition. For example, patients whose tumours are not infiltrated by effector immune cells are likely to benefit from the adoptive transfer of natural killer (NK) or T cells to boost infiltration of their tumors, which is an approach that is also currently being explored in HCC. 

Most forms of cancer immunotherapy that go beyond checkpoint inhibition are still at a preclinical or early clinical stage. Examples of such immunotherapies are chimeric antigen receptor (CAR-) T cells, allogeneic NK cells, and oncolytic viruses (Table 6). Currently, there are six registered phase 1 studies with CAR-T cells targeting Glypican 3 (GPC3) (NCT04121273; NCT04506983; NCT03198546; NCT02905188; NCT03884751; NCT03980288). There is one phase 2 study that is comparing treatment with invariant NKT cells and TACE with TACE alone (NCT04011033). Similarly, FT500, an allogeneic NK cell-line, and FATE-NK100, which are donor-derived NK cells, are being tested in phase 1 trials in various cancer entities including HCC (NCT03319459; NCT04106167; NCT03841110). After the oncolytic virus pexastimogene devacirepvec (Pexa-Vec) failed as second-line monotherapy in advanced HCC in the TRAVERSE phase 2b trial [39], it is now being tested in combination with nivolumab in a phase 1/2a trial (NCT03071094). However, the PHOCUS trial (NCT02562755; phase 3) [40], which studied the combination of Pexa-Vec and sorafenib, stopped enrolling patients prematurely due to the apparent lack of benefit in a planned interim futility analysis, highlighting the odds of failure for novel immune interventions during clinical development.

## 7. Outlook and Conclusions

**Figure 1:**
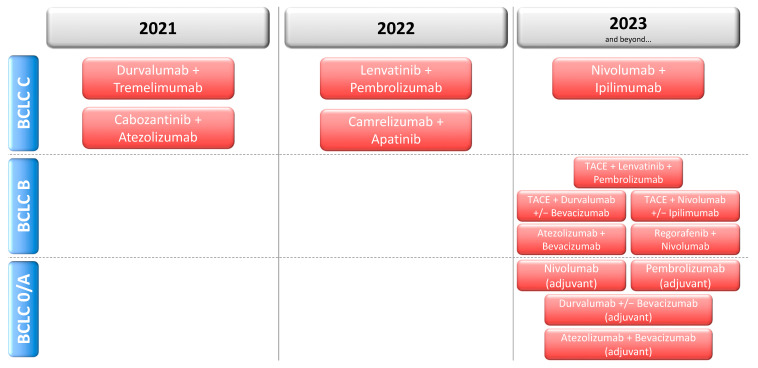
Expected years of availability of results from ongoing phase 3 clinical trials in HCC.

Systemic treatment for HCC has gained considerable momentum in recent years, but never has its outlook been as bright and diverse as today: The breadth of CTs evaluating systemic treatments as adjuvant treatment after curative surgery or ablation, as an addition to TACE, and as palliative first or second-line treatment is unprecedented. The coming years will see a host of data from phase 3 CTs (Figure 1), which have a high chance of bringing a profound change to the clinical management of HCC.

As of today, atezo/bev is the first and only immunotherapy with a proven benefit in HCC. It is noteworthy that all currently ongoing major CTs involve at least one immunotherapeutic agent. Further, such agents are not confined to advanced HCC any longer, but tested in earlier disease stages. Furthermore, sophisticated immune interventions such as CAR-T or CAR-NK cells have now entered clinical development and may expand the armamentarium for HCC in the long term.

Currently, the most prevalent approach is to combine a RTKI or anti-VEGF antibody with a checkpoint inhibitor. While none of the ongoing CTs uses atezo/bev as a comparator, the efficacy of such regimens will have to be compared to atezo/bev in the advanced HCC first-line setting. And this comparison goes beyond efficacy and needs to pay attention to the toxicity profile and quality of life data as well. Atezo/bev was better tolerated and the quality of life longer maintained compared to sorafenib in the IMbrave150 trial, [41] a relevant aspect for patients in the palliative setting. However, given the issues with inter-trial comparisons it will be difficult if not impossible to judge which first-line treatment is best. In addition, data on the sequence of treatments will be lacking making this an important clinical issue in the coming years. Taken together, all these developments make it appear very likely that the relevance of cancer immunotherapy for HCC will continue to increase.

In spite of the excitement around immunotherapy for HCC, it is fairly certain that not every patient will derive equal benefit from current and future systemic treatments. The reason for this lies in the heterogeneity of HCC, which is also reflected in its immune contexture. The composition of the immune contexture influences the prognosis of HCC [42,43,44] and can therefore be utilized for immunophenotypic classifications [43,45]. However, it is still unknown whether any of the proposed signatures or classifications are capable of predicting the response to immunotherapy. Though, first proofs-of-concept have been demonstrated: WNT/CTNNB1 mutations have been associated with resistance against checkpoint inhibition [46,47]. Furthermore, an interferon-γ-related gene signature that predicts the response to pembrolizumab has been reported [48], but this has not been shown for HCC. Other biomarker candidates such as tumor mutation burden, T cell repertoire clonality change, and gut microbioal diversity are being studied (reviewed in [49]). Further research is needed to develop practical and robust biomarkers that predict the response to treatment and that can be used under real-world conditions. This would allow clinicians to select the most effective systemic treatments for patients with HCC reducing potential harm from individually ineffective regimens.

Another weakness of the current landscape of CTs is that virtually all require an idealized type of HCC patient excluding patients with advanced liver disease, comorbidities and special conditions, which constitute the majority of patients in real-world practice. Recently, we have highlighted the neglect of such patients in CTs [50]. The currently prevalent design of CTs fosters a lack of data for these subgroups of patients perpetuating the challenge of choosing the best treatment strategy for them.

In conclusion, the current landscape of CTs for the systemic treatment of HCC looks highly promising. Cancer immunotherapy now has its place in the treatment algorithm of HCC, and it is likely that its reach will continue to grow. A wealth of exciting data from CTs can be expected in the coming years, which will hopefully provide HCC patients with better treatment options and improved prognosis.

## Figures and Tables

**Table 2:** Current clinicals trials on palliative systemic treatments in HCC.

Trial	Identifier	Phase	BCLC Stage	Treatment Arms	Primary Endpoint(s)	Setting
COSMIC-312	NCT03755791	Phase 3	B or C	Cabozantinib + atezolizumab Sorafenib Cabozantinib	PFS per RECIST 1.1 OS	First-line
LEAP-002	NCT03713593	Phase 3	B or C	Lenvatinib + pembrolizumab Lenvatinib	PFS per RECIST 1.1 OS	First-line
HIMALAYA	NCT03298451	Phase 3	B or C	Durvalumab Durvalumab + trevelimumab (2 regimens) Sorafenib	OS	First-line
CheckMate 9DW	NCT04039607	Phase 3	C	Nivolumab + ipilimumab Sorafenib or lenvatinib	OS	First-line
N/A	NCT03764293	Phase 3	B or C	Camrelizumab (SHR-1210) + apatinib Sorafenib	PFS OS	First-line
RATIONALE-301	NCT03412773	Phase 3	B or C	Tislelizumab Sorafenib	OS	First-line
GOING	NCT04170556	Phase 1/2	BCLC C	Regorafenib (monotherapy for the first 8 weeks) + nivolumab	Safety	Second-line
RENOBATE	NCT04310709	Phase 2	B or C	Regorafenib + nivolumab	ORR per RECIST 1.1	First-line
Bayer 19497	NCT03347292	Phase 1b/2	B or C	Regorafenib + pembrolizumab	Safety	First-line
N/A	NCT04183088	Phase 2	B or C	Part 1: Regorafenib + tislelizumab Part 2: Regorafenib + tislelizumab Regorafenib	Part 1: Safety Part 2: PFS per RECIST 1.1 ORR per RECIST 1.1	First-line
N/A	NCT04442581	Phase 2	B or C	Cabozantinib + pembrolizumab	ORR per RECIST 1.1	First-line
N/A	NCT03941873	Phase 1/2	B or C	Phase 1: Sitravatinib Sitravatinib + tislelizumab Phase 2: Sitravatinib Sitravatinib + tislelizumab	Phase 1: Safety Phase 2: ORR per RECIST 1.1	First- and later line

BCLC, Barcelona Clinic Liver Cancer; ORR, overall response rate; OS, overall survival; PFS, progression-free survival; RECIST, Response Evaluation Criteria in Solid Tumours.

**Table 3:** Current clinical trials combining or comparing systemic treatments with TACE.

Trial	Identifier	Phase	BCLC Stage	Treatment Arms	Primary Endpoint(s)	Setting
LEAP-012	NCT04246177	Phase 3	B	Lenvatinib + pembrolizumab + TACE TACE	PFS per RECIST 1.1 OS	First-line
EMERALD-1	NCT03778957	Phase 3	B	Durvalumab + TACE Durvalumab + bevacizumab + TACE TACE	PFS per RECIST 1.1	First-line
CheckMate 74W	NCT04340193	Phase 3	B	Nivolumab + ipilimumab + TACE Nivolumab + TACE TACE	Time to TACE progression OS	First-line
ABC-HCC	NCT04803994	Phase 3	B	Atezolizumab + bevacizumab TACE	Time to failure of treatment strategy	First-line
RENOTACE	NCT04777851	Phase 3	B	Regorafenib + nivolumab TACE	PFS per mRECIST	First-line

BCLC, Barcelona Clinic Liver Cancer; OS, overall survival; PFS, progression-free survival; RECIST, Response Evaluation Criteria in Solid Tumours; TACE, transarterial chemoembolization.

**Table 4:** Current clinical trials on adjuvant systemic treatments after surgery or ablation.

Trial	Identifier	Phase	BCLC Stage	Treatment Arms	Primary Endpoint(s)	Setting
CheckMate 9DX	NCT03383458	Phase 3	0 or A	Nivolumab Placebo	RFS	Adjuvant
KEYNOTE-937	NCT03867084	Phase 3	0 or A	Pembrolizumab Placebo	RFS OS	Adjuvant
IMbrave050	NCT04102098	Phase 3	0 or A	Atezolizumab + bevacizumab Active surveillance	RFS	Adjuvant
EMERALD-2	NCT03847428	Phase 3	0 or A	Durvalumab + bevacizumab Durvalumab Placebo	RFS	Adjuvant

BCLC, Barcelona Clinic Liver Cancer; OS, overall survival; RFS, recurrence-free survival.

**Table 5:** Current clinical trials on neoadjuvant systemic treatments.

Trial	Identifier	Phase	BCLC Stage	Treatment Arms	Primary Endpoint(s)	Setting
NIVOLEP	NCT03630640	Phase 2	A or B	Nivolumab + electroporation	Local RFS	Neoadjuvant & adjuvant
CaboNivo	NCT03299946	Phase 1b	N/A *	Cabozantinib + nivolumab + surgery	Safety	Neoadjuvant
N/A	NCT03337841	Phase 2	0 or A	Pembrolizumab + surgery/ablation	One-year RFS rate	Neoadjuvant & adjuvant
PLENTY202001	NCT04425226	Phase 2	N/A **	Pembrolizumab + Lenvatinib + liver transplantation No intervention + liver transplantation	RFS	Neoadjuvant

* locally advanced/borderline resectable HCC; ** HCC exceeding Milan criteria. BCLC, Barcelona Clinic Liver Cancer; N/A, not available; RFS, recurrence-free survival.

**Table 6:** Selection of current clinical trials on gene and cell-based systemic treatments.

Identifier	Phase	BCLC Stage	Treatment Arms	Primary Endpoint(s)	Setting
NCT02905188	Phase 1	C	CAR-GPC3 T cells	Safety	Palliative
NCT03980288	Phase 1	C	CAR-GPC3 T cells	Safety	Palliative
NCT04011033	Phase 2	C	iNKT cells + TACE	OS PFS DCR	Palliative
NCT03319459	Phase 1	C	FATE-NK100	Safety	Palliative
NCT03841110	Phase 1	C	FT500 (allogeneic NK cells)	Safety	Palliative
NCT03071094	Phase 1/2	C	Pexastimogene devacirepvec + nivolumab	Safety ORR	Palliative

BCLC, Barcelona Clinic Liver Cancer; DCR, disease control rate; iNKT cells, invariant natural killer T cells; NK cells, natural killer cells; ORR, overall response rate; OS, overall survival; PFS, progression-free survival; TACE, transarterial chemoembolization.

## Data Availability

Not applicable.
